# Injections and HIV in Rural Zimbabwe

**DOI:** 10.1371/journal.pmed.0020054

**Published:** 2005-02-22

Of the 40 million people worldwide with HIV, 30 million live in the developing world. By far the worst hit region is sub-Saharan Africa, where nearly four million children have lost one or both parents to HIV/AIDS since 2000. Is heterosexual transmission the driving force behind the HIV epidemic in sub-Saharan Africa? In a controversial debate, some researchers have suggested that other factors such as unsafe medical injection practices may also be to blame, and that by overlooking, and even suppressing, analysis of this possible route of transmission, the current focus on preventing sexual transmission may be misguided. In this month&apos;s *PLoS Medicine*, Ben Lopman and colleagues argue that although it is right to criticize the lack of evidence on unsafe medical injection, field data are hard to collect. They note that in the only published study addressing this issue, Kiwanuka and colleagues found no link between unsafe injections and HIV spread in rural Uganda. In an effort to “inform the debate” further, Lopman and colleagues looked at the association between HIV and unsafe injection practices in rural Zimbabwe.[Fig pmed-0020054-g001]

**Figure pmed-0020054-g001:**
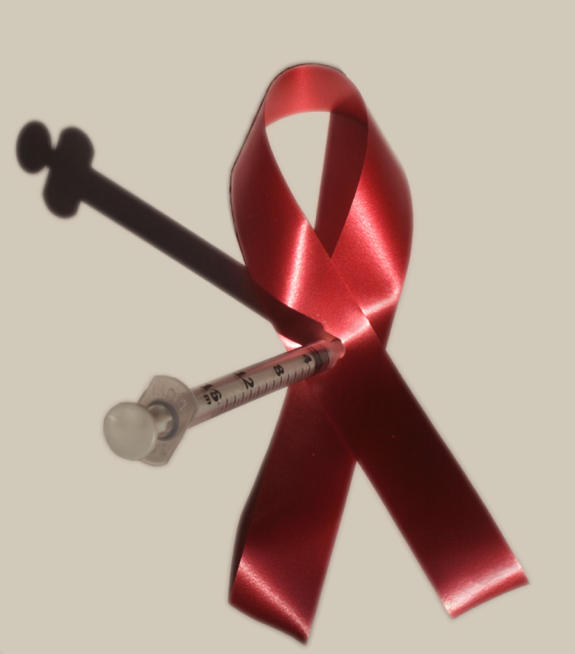
Are medical injections an important cause of HIV in rural Africa?

The team analyzed data from adults in Manicaland, a rural part of Zimbabwe, who were taking part in the Manicaland HIV/STD Prevention Study. In 1999 and 2000, eligible patients were tested for HIV and surveyed (86.7% were HIV negative at the start of the study), and were followed up three years later. The team collected survey data on injections in the patients, who were male and female adults aged 15 to 54 years old, and tested for an association between injection exposure and HIV infection. In 2002 and 2003, 505 of the men and 1,342 of the women, representing a 69.7% follow-up, were again interviewed and tested for HIV infection. Of these, 40% reported having had an injection or needle prick during the study period. A total of 67 patients developed HIV during the study; of these 13 (19%) said they had not had sex during the study period and 40 (60%) said they had not had an injection. The statistical analysis found no significant association between injections and HIV infection in men or women.

Patients who had HIV when the study began did not have higher rates of injections. Instead, injections were highly associated with childbirth and pregnancy. But since HIV–positive women have reduced fertility, a reduction in the use of maternal services may partially explain why injections were not more common in these HIV-positive patients. In this study, the strongest predictor of HIV infection was symptoms of sexually transmitted disease.

Despite problems of recall bias and under-reporting of sexual activity—a particularly difficult problem in studies in Africa—sexual behavior is consistently linked with HIV incidence. Where does this leave the debate over injections in Africa? Certainly, for this community, they do not seem to be a major source of HIV infection, and local policy-makers would therefore do best to concentrate on the prevention of sexually transmitted infections.

